# Special Issue: Genomic Data in Pathogenic Fungi

**DOI:** 10.3390/jof4010040

**Published:** 2018-03-20

**Authors:** Theo N. Kirkland

**Affiliations:** Departments of Pathology and Medicine, School of Medicine, University of California, San Diego, CA 92037, USA; tkirkland@vapop.ucsd.edu

This special issue, “Genomic Data in Pathogenic Fungi,” focuses on the genomics of human and plant pathogens. Efforts like this are important because so little information about these organisms is available. There are fewer than 350 publications in the PubMed database reporting genomic data of filamentous fungi that are primary human pathogens, such as *Histoplasma capsulatum*, *Coccidioides* spp., *Blastomyces* spp. or *Sporothrix* spp., compared to more than 20,000 publications about *Saccharomyces cerevisiae*. Although there are many reasons for this, the disparity indicates the need for more information about primary pathogenic fungi as well as model fungal organisms. Special issues such as this attempt to address this disparity.

Castiblanco et al. [1] describe pathogenesis genes in *Fusarium colmorum* with respect to rye head blight and provide vital information about fungal pathogenesis. The authors use a combination of quantitative trait locus mapping and SNP calling to identify an SNP associated with the ability to cause disease in rye. This SNP, upstream of the secreted cutinase, is required to penetrate plant cuticles and presumably affects transcription of the cutinase gene, although transcription studies in such highly pathogenic and less pathogenic strains will be important to confirm these data.

Understanding the fungal cell wall is a critical component of understanding fungi and their pathogenic potential. A large-scale comparison of the cell-wall-related components of multiple pathogenic fungi is one way to understand them. Muszeska et al. [2] identify and classify cell-wall-related proteins of 24 human fungal pathogens. An evolutionary history of these cell-wall-related proteins is determined via analysis of their phylogenetic relationships. Most of these proteins are enzymes involved in the synthesis or degradation of complex cell wall carbohydrates, especially chitin. The identification of 4000 fungal proteins is expected to lead to novel insights on the synthesis of cell wall components. 

Non-protein encoding genomic transposable elements are important for epigenetic phenomena. All fungi contain transposons, but the number and type of such transposons vary substantially between taxa. Kirkland et al. [3] characterize the transposons in *Coccidioides* spp., important human pathogens in the desert regions of the new world. LTR/Gypsy transposons are the most common in *Coccidioides* spp.; most of them have degenerated and lack some of the coding regions required for transposition. Although most transposons are found in gene-poor regions, the genes that are flanked by transposons tend to be expressed at an average level that is lower than that of all genes, especially in *C. immitis*. Genes coding for protein phosphorylation tend to be over-represented in the genes flanking transposons in both *C. immitis* and *C. posadasii*. The biological significance of these observations is not yet clear.

Dealing with large amounts of data and extracting biologically meaningful insights is always a challenge. Basenko describes FungiDB [4], an online resource containing genomic information of almost 100 fungi. FungiDB contains many types of information and tools to link this information together. Some of the available data include genomic and transcriptomic information, protein sequences, and proteomic information, phenotypes, and links between species, such as their orthology and synteny. Examples of the analysis tools and methods for using them are shown. FungiDB is demonstrated to be a very useful resource for researchers in this field.

Improving our understanding of primary pathogenic fungi will require thoughtful experiments and data analysis. It is hoped that this special issue can make a small contribution to this effort. 
